# The Second Annual Meeting of the International Hahnemannian Association

**Published:** 1882-09

**Authors:** J. B. Gregg Custis

**Affiliations:** Secretary


### THE SECOND ANNUAL MEETING OF THE INTERNATIONAL HAHNEMANNIAN ASSOCIATION.

The Second Annual Meeting of the Association was held at Indianapolis, Indiana, June 13th, 1882, and was called to order by the President, Dr. C. Pearson.

The Secretary, Dr. James, being absent, on motion of Dr. Gregg, Dr. Custis was chosen Secretary *pro tem.*

The President then addressed the Association on "The Duties of the Hour." On motion of Dr. Gregg it was ordered that the address be printed in pamphlet form and distributed to the profession.

The reports of the Secretary and Treasurer were received and approved, the latter showing a balance of sixty dollars on hand.

A letter from Dr. Lippe was read regretting his inability to be present.

The President appointed Drs. Gregg, Mills and Cranch as an acting Board of Censors, who reported favorably upon the following applications for membership, which were accompanied by fees as required by the By-Laws, viz.: Dr. Benj. Ehrman, Cincinnati, Ohio; Dr. Fred. Ehrman, Cincinnati, Ohio; Dr. C. H. Lawton, Wilmington, Del.; Dr. C. E. Prentiss, Washington, D. C.; Dr. W. Sam'l Arrowsmith, Wateringbury, England; Dr. Jas. Henry Payne, Boston, Mass.; Dr. De Forest Hunt, Brooklyn, N. Y.; Dr. Danl. W. Clausen, Auburn, N. Y.; who were elected, severally, by ballot.

The Censors also reported favorably on the application of the following, who were elected, subject to compliance with the rules of the Association, viz.: Dr. Chas. F. Millspaugh, Binghamton, N. Y.; Dr. Mahlon Preston, Norristown, Pa.; Dr. T. S. Hoyne, Chicago, Ill.; Dr. E. P. Hussey, Buffalo, N. Y.; Dr. Fred. S. White, Exeter, England; Dr. Geo. Dunn, London, England; Dr. Wm. Bradshaw, Folkstone, England; Dr. Edw. Mahone, Liverpool, England; Dr. Arthur de Noe Walker, Ovington Gardens, England.

A letter, regretting his inability to be present, was received from Dr. Ballard, who suggested the publication of a directory of the members for the use of the Association; also one from Dr. Swan, presenting a seal and a number of certificates of membership.

The following Amendments to the Constitution and By-Laws, which were proposed at the preceding meeting, were considered and adopted, viz.:

To Article I of the Constitution: "This Society shall be known as the International Hahnemannian Association, the objects of which are fully set forth in its declaration of principles."

To Article III of the By-Laws: "If an applicant for membership be not elected by the first ballot, he may, upon a majority vote, if he so desires, receive a second ballot at the next Annual Meeting of the Association."

The following resolutions proposed by Dr. Berridge were adopted, viz.:

*Resolved*, 1. That provings on healthy persons constitute the basis of our materia medica, but that, nevertheless, following the example of Hahnemann and his early disciples, we may cautiously supplement these by provings on the sick, and by frequently verified clinical experience.

2. That the Secretary be instructed to send a copy of the *Transactions* of the year 1882, with the resolutions, by-laws and constitution of the Association, together with a complete list of the members to the various continental homœopathic journals, with a request that they call the attention of their readers to them, with a view to enrolling new members.

3. That the Association publish, annually, a Hahnemannian Directory, consisting exclusively of members of the Association, arranged first alphabetically, under the names of the members the full address and all the titles of each being given; and secondly, according to their localities.

4. That every year in the number of THE HOMŒOPATHIC PHYSICIAN which contains the year's transactions, a complete alphabetical list of the members with the date of their joining the Association, be published.

5. That every member be entitled to add after his degrees of M. D., etc., etc., M. I. H. A.

6. That the resolutions, by-laws and constitution of the I. H. A. be annually published with the *Transactions*, showing additions or alterations made from time to time.

A letter from Dr. Nichols was read, expressing regret for his inability to be present, and proposing the following resolution, viz.:

*Resolved*, That THE HOMŒOPATHIC PHYSICIAN be no longer the organ of the International Hahnemannian Association, since its editors refused to publish homœopathic cures made with nosodes.*

The resolution was generally debated, with expressions of regret that any members should be restricted in their articles, which were so full of interest to the Association, and being unwilling to believe that the resolution was necessary, it was laid on the table.

The following resolution was adopted:

*Resolved*, That all papers accepted by the Association and Publication Committee shall be published in the proceedings of the Association.

The Association then adjourned to Wednesday, 9 A. M.

### SECOND DAY.

The Association met pursuant to adjournment, the President in the chair.

The reading of papers was postponed until Thursday, in order that all business might first be completed.

On motion of Dr. Gregg, it was unanimously resolved that the thanks of the Association be tendered to Dr. Swan, for his generous gift of a seal and certificates of membership, the design and execution of which are so eminently satisfactory.

---

* THE HOMŒOPATHIC PHYSICIAN has only *one* editor, who assumes all responsibility. And, as it is *generally* the editor's business to say what matter his journal will publish, we cannot clearly discern what the I. H. A. has to do with the matter. We publish such papers as are, in our humble judgment, best calculated to teach true Homœopathy, and as far as our memory goes, we do not recollect having refused any "homœopathic cures" that *aided* in this purpose.—Editor H. P.

The following officers were then elected for the ensuing year, viz.:

*President*, C. Pearson, M. D., Washington, D. C.; *Vice-President*, J. P. Mills, M. D., Chicago, Ill.; *Secretary*, J. B. Gregg Custis, M. D., Washington, D. C.; *Corresponding Secretary*, E. W. Berridge, M. D., London, England; *Treasurer*, Edward Cranch, M. D., Erie, Pa.

*Board of Censors.*—T. F. Pomeroy, M. D., *Chairman*, Baltimore, Md.; Benj. Ehrman, M. D., Cincinnati, O.; Samuel Swan, M. D., New York, N. Y.; T. P. Wilson, M. D., Ann Arbor, Mich.; C. H. Lawton, M. D., Wilmington, Del.

The President appointed as Chairmen of Bureaus for 1883, the following, viz.:

*Bureau of Clinical Medicine.*—R. R. Gregg, M. D., Buffalo, N. Y.

*Bureau of Materia Medica and Provings.*—O. P. Baer, M. D., Richmond, Ind.

*Bureau of Obstetrics, etc.*—J. R. Haynes, M. D., Indianapolis, Ind.

*Bureau of Surgery.*—H. I. Ostrom, New York, N. Y.

The Association then adjourned to 9 A. M. of Thursday.

### Third Day.

The Association met pursuant to adjournment, the President in the chair.

Papers were read by Drs. Baer and Custis, and generally discussed until 12 M., when the Association took a recess until 2 P. M.

Reassembling at the appointed time, papers by Drs. Haynes, L. B. Wells, Bell and Nichols were read and discussed.

The following resolutions, proposed by Dr. C. E. Prentiss, were considered and adopted, viz.:

*Resolved*, 1. That the officers of the Association shall constitute the Executive Committee, and it shall be their duty to make arrangements for the meetings of the Association; to attend to all business not otherwise provided for, and to perform such other duties as by vote of the Association may be assigned to them.

2. That at each annual meeting, the Association shall elect a Board of Censors, consisting of five members, three of whom shall constitute a quorum, who shall receive and examine the applications of candidates for membership, and report to the Association for election, the names of such as may be found qualified.

3. That each bureau shall consist of not less than seven members, the additional members to be appointed by the chairman, who shall, as soon as possible after his appointment, organize his bureau, which shall select a special subject of inquiry, to be reported upon at the next annual meeting of the Association.

Dr. Pearson offered the following amendments to the By-Laws, to be acted on at the next meeting of the Association, viz.:

That Article I read as follows: "This Association shall meet annually at such time and place as a majority of its members may advise."

Alterations in Article III, as follows: "Applicants for membership shall send in their names and recommendations to the Chairman of the Board of Censors; the election shall be by ballot, and a two-thirds majority of the members present at said annual meeting is necessary to elect."

Alterations in Article IV, as follows: "Every member on his or her election, shall pay to the Treasurer the sum of two dollars, and one dollar annually thereafter."

"Article VIII. Inasmuch as this Association is international in its character, that for all officers, foreign members be permitted to vote by proxy."

The Executive Committee was instructed to make all necessary arrangements for the next meeting, and to prepare an order of business, and send a copy to each member.

The Secretary was instructed to return rejected applications for membership, with the reasons for such rejection, as stated by the Board of Censors.

The Association then adjourned *sine die.*

J. B. Gregg Custis, M. D., *Secretary.*

Note.—The sessions devoted to the discussion of papers were exceedingly pleasant and profitable, and the Secretary expresses the hope that the Association may, at its next meeting, secure the services of a stenographer, that the profession may have the advantage of all such discussions, which brought out the results of much valuable experience.

*Secretary.*

